# Children’s technologies of the self within neoliberal governmentality at the educational transition to *Gymnasium* in Zurich

**DOI:** 10.1080/14733285.2023.2237925

**Published:** 2023-07-25

**Authors:** Lara Landolt, Itta Bauer

**Affiliations:** Department of Geography, University of Zurich, Zurich, Switzerland

**Keywords:** Technologies of the self, self-governance, neoliberal governmentality, student voice, education, Switzerland

## Abstract

Over the last two decades, research in children’s geographies and governmentality studies have contributed significantly to the study of children’s experiences in neoliberal educational contexts. This paper furthers this debate by examining the ways children govern and are governed within the neoliberal governmentality at the educational transition to *Gymnasium*: the only school that offers a direct path to university education within the state-funded school system in Switzerland. Drawing on an ethnography with eight students aged 13–15 during their preparation for the selective entrance examination to *Gymnasium* in Zurich, this article makes two points: Firstly, it demonstrates how Zurich’s education system thrusts students into taking individual responsibility for their educational success at this transition*.* Secondly, the article draws on Foucault’s later work to explore the particular ‘technologies of the self’ that children adopt coping with this individualized responsibility. This paper argues that these technologies reveal insights into the neoliberal governmentality of this educational transition. Finally, the article argues to critically examine children’s technologies of the self to understand their relationships with the education systems they navigate. This line of inquiry serves as a pathway to answer and expand earlier calls to grant children an active voice in research on education.

## 1. Introduction

**‘**When I sit in bed like this, I think: What are you doing? What are you doing? You want to make it to *Gymnasium*! You have to study!’, Aiza^1^ tells us as I (Lara, first author)^2^ am sitting with some of her fellow students in the hallway during a break. Emily and Liah nod in agreement. During the Christmas break, she would have been ‘so good’ at studying, but now it had changed: ‘somehow, I just can’t do this anymore’, Aiza explains. Hence, she would like to do things differently during the next holidays, which begin in a week’s time. She had agreed with her mother that she will go for walks more often this last holiday before the Central Entrance Examination and will no longer study 16 h a day. Liah and Emily do not seem to agree with Aiza’s new attitude, because they start to explain that Aiza had always given them a lot of motivation to study as much as she does. Liah tells us: ‘She is such a motivation for me!’. ‘You’re so good, Aiza!’, adds Emily. ‘Don’t give up now’, Liah says. Aiza admits that she just doesn’t want to feel down again and that she would need to think of a new ‘strategy’ for the holidays. (Fieldnote taken during a break at a private exam preparation course in February 2022)

Aiza is an aspiring 14-year-old secondary school student preparing for the standardized Central Entrance Examination (CEE) to *Gymnasium* in Zurich. Zurich’s educational model includes two transitions to *Gymnasium*, both regulated through a CEE: The first and more popular one takes place after six years of primary school (students aged 11–12) and the second transition, is possible after year eight or nine (aged 13–15). This paper exclusively focuses on the latter transition. In 2022, just 6.9% of all eighth – and 3.6% of all ninth-year secondary school students successfully transitioned to a *Gymnasium* in the Canton of Zurich. This percentage is complemented by another 15.5% of students, who transitioned to *Gymnasium* already after year six that year (BISTA 2023). After completing education at state schools, the majority of students in the Canton of Zurich move on to a vocational education and training school for an apprenticeship. Only a minority of students enter a *Gymnasium* if they have passed the CEE. *Gymnasium* is the only general education school that offers a strong academic profile and a direct path to university education. Hence, it is primarily academically ambitious students that sign up for the entrance test to this state-funded school track. For Aiza, *Gymnasium* presents the direct route to her dream of becoming a lawyer. As this transition is highly selective, the months prior to the CEE are an intense period of preparation for those students who aspire to attend a *Gymnasium*. In the hope of increasing their chances of a pass, students preparing for the CEE increasingly turn to fee-based private tutoring (Hof and Wolter 2014) and other forms of support provided by their schools or private, nonprofit initiatives for children from families with low socioeconomic status, which a few students such as Aiza are able to visit. Aiza’s relentless efforts to prepare herself for the CEE despite revealing that she ‘just can’t do this anymore’ not only illustrates the pressure some children experience at this transition but also echoes the broader debate on neoliberalization in education. By repeatedly telling herself ‘you want to make it to *Gymnasium*! You have to study!’, Aiza exemplarily mirrors the effects that a neoliberal ‘politics of aspiration’ (Raco 2009) may have on students: they see themselves forced to act as self-reliant, self-responsible entrepreneurial citizens (Brown 2011, 8).

This study builds on a wealth of research analysing neoliberal trends in education and elaborating how children are indirectly affected by these developments in various educational and geographical contexts (e.g. Bradbury 2019; Davidson 2008; Grant 2017; Holloway and Kirby 2020; Holloway and Pimlott-Wilson 2011; Pimlott-Wilson 2019). Our paper furthers this research in two respects: (1) Geographically, we move beyond the Anglophone pivot in the field. (2) Conceptually, we read our ethnographic data through a governmentality perspective that focuses on the complex realities of children living through an educational transition characterized by high selectivity. This paper draws on Foucault’s later work on ‘governmentality’ and ‘technologies of the self’ (1988) to show how children who are dedicated to entering a *Gymnasium* are governed by subjectivizing themselves through appropriating certain ‘self-techniques’ while preparing for the CEE.

Drawing on in-depth data from a five-month ethnography with eight students aged 13–15 aspiring to transfer to *Gymnasium*, our paper follows two distinctive research objectives which combine to produce innovative insights into children’s ‘technologies of the self’ within neoliberal governmentality: Firstly, we aim to show how Zurich’s education system thrusts students into taking individual responsibility for their educational success at the transition to *Gymnasium*. Secondly, we want to demonstrate how children within this educational context are governed and govern themselves by appropriating certain ‘technologies of the self’. Applying this Foucauldian concept, we focus on children’s ways of disciplining, caring for, and reflecting on the self to transform themselves into an idealized subject ready for the CEE. By focusing not only on the ways children are governed in educational contexts, but specifically on their personal ways of governing themselves, we argue for the analysis of the complex entanglements of children within neoliberal governmentality and for giving children an active voice in this debate (cf. Nguyen, Cohen, and Huff 2017).

In the next section, we review the existing literature on neoliberal governmentality in education with a particular focus on research into children’s experiences. Then, we describe Zurich’s educational transition to *Gymnasium*. Section four depicts our data and ethnographic method. We present the findings of our research in section five and conclude with a short reflection on this paper’s implications for further educational research in children’s geographies and governmentality studies.

## 2. Neoliberal governmentality within education

Since the 1980s, formal education and many diverse learning environments worldwide have increasingly displayed a shift towards neoliberal vocabulary, thought, and practice (Dahlstedt and Fejes 2019; Davies and Bansel 2007; Themelis 2020). An array of phenomena have been labeled as neoliberal and involve ‘aspects of deregulation, privatization and withdrawal of the state from social provision’ (Schwiter 2013, 153). In education, the introduction of international student assessment tools exacerbated cross-national competition among state-funded education systems and nudged education offices to improve the results of their students’ competencies (Goldstein 2004). Education systems worldwide have shown trends towards privatization and the development of a supplementary education industry (Bray 2017). These tendencies are often discussed controversially as private supplementary education plays a central role in educational systems where exams work as an incisive guideway to students’ educational trajectories, such as in Zurich (Bauer and Landolt 2022) and elsewhere (Holloway and Kirby 2020; Phillippo 2019). Next to commercializing and privatizing access to education, neoliberalism entails the implementation of education policies that promote the idea of an idealized self-reliant, self-responsible, entrepreneurial, and aspiring citizen (e.g. Brown 2011; Davidson 2008; 2015; Grant 2017; Hangartner et al. 2022; Pimlott-Wilson 2019). Researchers have observed that neoliberalism is not merely a hegemonic mode of socioeconomic and political organization engrained in policies and discourse ‘out there’ (Holloway and Pimlott-Wilson 2012; Laval and Dardot 2013; Schwiter 2013). Neoliberalism extends to all areas of life ‘in here’: ‘in the head, the heart and the soul’ of people (Ball 2016, 1047). This not only affects individuals but also fundamentally changes the ways we interact with each other (Massey 2013).

Adapting this argument to education, we are rewarded with a narrative that accounts not only for broad-scale accounts of neoliberal processes and policies but also captures the effects these restructured environments have on subjects, including children navigating education systems. For example, Mitchell argues that in today’s market-mediated, globalized context, education and learning are regarded as developing one’s human capital, which epitomizes the idea of (wo)man as a calculating, self-investing, rational *homo economicus*, a subjectivity that Mitchell terms the ‘entrepreneurial child’ (2018, 25). Similar arguments have been elaborated by Bradbury (2019) who problematizes the English primary school’s ideal of the student as an ‘entrepreneur of the self’ or by Morrin (2018) discussing the ways ‘entrepreneurial character’ is reproduced, ‘refused’, and negotiated in an English secondary school. Davidson’s ethnographic fieldwork within a US high school reveals the extent to which students reflect a neoliberal politics of ‘marketing the self’ that shapes student’s forms of self-discipline, self-definition, and aspiration (2008, 2015). Davidson shows how students’ ways of categorizing their own and others’ educational successes and failures depend on particular values and traits of meritocracy and incorporated competitiveness (2008, 2815). Studying Chicago’s high school admittance policy, Phillippo (2019) discusses the ways educational structures and discourses influence young people’s lives and self-perceptions. Phillippo shares our empirical focus on the interplay of a highly selective entrance examination and a socially accepted discourse on the responsibilization of students for a successful transition. Phillippo and Griffin (2016) not only show how Chicago students are made responsible for their choices but also how students view educational opportunities through a merit-based perspective, even though their school admissions diverged along lines of social privilege.

Studying the effects that neoliberal education policies impose on children, some researchers have turned towards the Foucauldian concept of ‘governmentality’ (e.g. Peters et al. 2009). According to Foucault, neoliberalism presents itself in government practices (*gouverner*) and rationalities (*mentalité*), together termed governmentality, which operate through an array of techniques by which both individuals and states govern and are governed along neoliberal lines of thought (Foucault 2008). This perspective has proven valuable because it moves beyond the study of individuals’ behaviors and aspirations by including the tools, practices, and cultural contexts which subjects use. This follows from the fact that neoliberalism, as a form of governmentality, not only manages individuals’ actions through technologies of domination (Foucault 1977), but also directs how people conduct their own behavior through ‘technologies of the self’ (Foucault 1988, 19). As Foucault states,

technologies of the self . . . permit individuals to effect by their own means or with the help of others a certain number of operations on their own bodies and souls, thoughts, conduct, and way of being, so as to transform themselves in order to attain a certain state of happiness, purity, wisdom, perfection, or immortality. (Foucault 1988, 18)

In other words, technologies of the self are the ways individuals turn themselves into subjects through actions, such as writing, exercise, and meditation to transform themselves into a desired state of being. For Foucault, governmentality lies within the interactions ‘between the technologies of domination of others and those of the self’ (1988, 19), so that all individuals’ behaviors are the effect of the interplay between these two technologies (von Felden 2020, 53). Governing, according to Foucault, not only involves the suppression of subjectivity through technologies of domination but equally promotes and thrives from the invention of ‘technologies of the self’ that work towards the greater goals of government (Lemke, Krasmann, and Bröckling 2000, 29). Within neoliberal governmentality, individuals turn into ‘docile subjects’ that are tightly governed by neoliberal rationality yet simultaneously define themselves as ‘free’ (Davies and Bansel 2007, 256). It is thus the study of the points of contact between the technologies of domination of others and those of the self that allows a nuanced analysis of neoliberal mechanisms of power (Lemke, Krasmann, and Bröckling 2000, 30).

Governmentality perspectives have proven useful in research that has examined a plethora of practices including the introduction of crime prevention programs in German schools (Schreiber, Stein, and Pütz 2016) and social relationships in UK school dining rooms (Pike 2008). Of particular interest to us are studies that examined neoliberal governmentality affecting children, such as in portfolio and statutory assessments (Bradbury 2019; Carlson 2009), standardized testing in the US (Kelly 2019), the promotion of a self-governing imperative within German and Swiss classrooms (Hangartner et al. 2022), and young people’s transitions from school to work in Germany (Hörschelmann 2008). These studies reveal how students’ subjectivities within neoliberal governmentality underlie normative discourses that promote an idealized student. Researchers have shown how these normative discourses relate to categories, such as class and gender, that render some students ‘impossible learners’ and diminish their chances for educational success (Youdell 2006), while promoting others as ‘ideal’ students that make ‘good choices’ (Bradbury 2019, 321). These idealizations are constituted ‘by the image of the individualized, autonomous and self-possessed political subject’, which within neoliberal governmentality is ‘urged and educated to bridle one’s own passions, to control one’s own instincts, to govern oneself’ (Rose 1999, 1, 4).

## 3. The selective transition to Gymnasium in Zurich and its education market

The proportion of students who graduate from *Gymnasium* in Switzerland rose from a 4% in the 1960s to a stable 20% since the 2000s (Bauer 2018; R. Becker and Zangger 2013, 428) and is regulated through an array of selection tools (SKBF 2018, 145). In comparison to other Swiss Cantons, the transition to *Gymnasium* in the Canton of Zurich, where the data presented in this article was collected, is particularly competitive. This is shown, among other things, by the rising number of parents with a high or medium occupational status and the above-average expectations of parents of their children’s educational trajectories (Herzing et al. 2022). Despite these socioeconomic changes that took place during the last decades, the proportion of students who graduate from *Gymnasium* has been held at a steady 20% (c.f. Criblez 2014; Pfister 2022). Zurich follows a restrictive admission procedure that is regulated through a standardized CEE, followed by a probationary period to confirm or correct the admission decision. Prior to introducing the standardized CEE in 2007, to assess students’ performances more consistently, each *Gymnasium* organized its own entrance test with similar acceptance rates (Bauer 2018).

Scholars have argued that the competition this educational transition produces rests in ‘neoliberal performance fantasies’ (Bauer and Landolt 2022, 18). This standardized transition to *Gymnasium* feeds a growing market of private supplementary education: First, private educational entrepreneurs and self-employed tutors serve the demand for small-group preparation courses and individual training for CEE. It is shown that the use of such fee-based supplementary education positively correlates with low transition quota to *Gymnasium* which is an indication of the high competition for limited places at *Gymnasium* in Zurich. As Hof and Wolter state: ‘Simply being “good” is not a guarantee to enter a *Gymnasium*, you have to be better than the others’ (2014, 10, own translation). The costs for such preparatory courses range from CHF 540 for a handful of lessons to CHF 5500 (approx. GBP 4885) for a whole semester (Kaffarnik and Stoll 2020). Second, state schools provide free-of-cost extracurricular preparation courses for a two hours per week for one semester (Bildungsdirektion Zürich 2012). These courses differ from school to school in their format, prerequisites, teachers, and quality (Kaffarnik and Stoll 2020). And third, private initiatives, such as ‘Chance4You’, offer intensive free-of-cost CEE preparation courses to a small number of selected students from families with low socioeconomic status as they seek to provide more equal chances at this educational transition (Bauer and Landolt 2022; Landolt and Kaegi 2020). This is because students from socioeconomically less privileged families are significantly underrepresented at *Gymnasium* (SKBF 2018, 159) and are disadvantaged by their teachers’ biased recommendations that discourage them to sign up for the CEE (Neuenschwander 2014) as well as by the socioeconomic composition of their school-districts (Dlabač, Amrhein, and Hug 2021). The preparation courses of state schools and private initiatives grew out of the intention to alleviate the unequal preparation conditions for students. They counter the ‘trend towards putting a price tag on preparing students for successful transition to *Gymnasium* and thus commodifying and privatizing this educational landscape’ (Bauer and Landolt 2022, 13).

## 4. Methods and data

This article builds upon an extensive collection of data Lara gathered within a five-month long ethnography with eight students aged 13–15 during their preparation for the CEE in Zurich between October 2021 and March 2022. Conducting this ethnography on the selective transition to *Gymnasium* produced in-depth data on the many discourses and practices that constitute the social reality of children living through this important period. The rich ethnographic data is complemented by interview data from all ethnography participants (Aiza, Caliyana, Eli, Eva, Madison, Maya, Nina, Rhea), their teachers and parents as well as four peers and friends (Céline, Jen, Hannah, Sam) of the ethnography participants that offered deeper insights into group dynamics. Lara visited state-funded preparation courses and Chance4You in early October 2021, where she sought participants for the research project. Eight students agreed to participate in the ethnographic study and comprised a mixed sample concerning socioeconomic status, migration background, school-district and CEE preparation program. The students that participated in this ethnography primarily included children with high ambitions that visited a fee-based preparation course or tutor (Eli, Eva, Maya, Nina, Rhea, Jen, Hannah) or Chance4You (Aiza, Caliyana, Céline, Sam). In most cases, they additionally visited their secondary school’s preparation course. It is important to note that Lara met other students, next to Madison, who did not attend a private CEE preparation course. For all participants anonymity has been maintained. The study is part of a wider research project and received approval from the Ethics Commission of the University of Zurich (number 21.4.13).

The data presented here was derived from fifty ethnographic fieldnote entries documenting participant observations while accompanying the children during their everyday lives in the months leading up to the CEE. Participants were observed at a variety of places: in private CEE preparation classes, at school, at information events for the various *Gymnasia*, at home, and at places where participants were hanging out after school. These observations were collected over the course of five months and each meeting took between two to five hours. Further, we draw upon nine semi-structured interviews (Longhurst 2010) with the participating children within the five months leading up to the CEE, six episodic interviews (Flick 2000) with the ethnography participants shortly after the results of the CEE were published in March 2022 and 12 semi-structured interviews with the teachers and parents of the ethnography participants. Lara analysed the complete data corpus with a grounded theory coding process following Charmaz (2006).

## 5. Findings

Two overarching themes resulted from the analysis, which will be presented in two corresponding parts: Firstly, individualization of responsibility for aspiring students has emerged as a central element in the governance of Zurich’s educational transition to *Gymnasium.* The trajectory to apprenticeship overshadowed the educational priority of students aspiring to transfer to *Gymnasium* after year eight or nine. Secondly, coping with this individualized responsibility, the children preparing for the CEE adopted certain ‘technologies of the self’ that showed themselves in children’s diverse ways of disciplining, caring for, and reflecting on the self. The article discusses these technologies arguing that they reveal insights into the neoliberal governmentality which this educational transition contributes to.

### 5.1. A system that individualizes responsibility for educational success

Every year in autumn, each *Gymnasium*, colloquially referred to as the ‘Gymi’, provides information about themselves at an event for interested students and their parents. In the autumn of 2021, Lara joined Nina and two of her friends for the information event about Nina’s preferred *Gymnasium*. For Nina, Gymnasium presented the direct route to her dream of becoming a teacher. During that evening, we listened to the headmaster presenting the *Gymnasium* in a light of excellence: The *Gymnasium* has a ‘beautiful library’, buildings with ‘award-winning architecture’, and plenty of ‘cultural activities’ for ‘personal development’. In a similar event, another headmaster explained that.

the *Gymi* is strict, and there is pressure, and not everyone is up for it. You have to want to ‘conquer’ education . . . if you see yourself in that, and you want to do that, then you are at the right spot at *Gymnasium*. (Fieldnotes, November 2021)

Most events Lara visited cultivated an image of the ideal student as persistent and independent, thus placing responsibility on the children. As one headmaster told the children in the audience: ‘Here, mummy and daddy can’t guide you anymore’. Talk of the ideal student propagated at the transition to *Gymnasium* was largely absorbed and reproduced by the students that aspired to pass the CEE. As Eva, one of Nina’s friends, told Lara:

You should be able to work in a concentrated way and study independently without anyone telling you. And you also have to be really ambitious and never give up. And you need to spend all your time making sure that you work well. (Interview, March 2022)

Eva’s understanding of the ideal student who is fit for the CEE underlies a notion of self-responsibilization that echoes in her ways of addressing ‘the ideal self’ in virtues, such as being concentrated, independent, ambitious, persistent, and even self-controlling. For many children within this study, the CEE and the virtues it connotes provoked feelings of stress and frustration. For example, Céline sometimes referred to the CEE as ‘this stupid exam’ and Eli would often describe himself being stressed and not sleeping well during the months leading up to the CEE. Despite the CEE ‘dragging him down’, Eli portrayed the preparation time for the CEE in a positive light: ‘how badly you want it’ would show at the CEE and hence confirm who would deserve to pass the CEE. For many students, the CEE preparation was ‘a matter of everyone preparing for themselves’ or ‘everyone’s own thing’, as Eli put it. Accordingly, students rarely shared information with their peers about how they actually prepared for the CEE outside of school.

Our data suggests that this process of individualizing children’s responsibility for educational success at the transition to *Gymnasium* stems from the structure of Zurich’s education system: Not only do the majority of secondary school students move on to a Vocational Education and Training school (VET) for an apprenticeship after year nine but the more popular track to the *Gymnasium* already starts off from year six after primary school. This way, the transition to *Gymnasium* after year eight or nine becomes sidelined, which shifts responsibility towards the ambitious students. In contrast to other competitive testing environments such as in Chicago (Phillippo 2019), secondary schools in Zurich do not track students’ performances, nor is it important for the schools that their students continue their education at *Gymnasium*.

Instead, our data suggests that at secondary schools, the trajectory to apprenticeship plays a much more prevalent role. When Lara was accompanying students through their days at school, finding apprenticeships was one of the most frequently discussed topics. Many of the classrooms were decorated with flyers about possible apprenticeships. Several classrooms had posters showing how far every student had come in their search for an apprenticeship. During one visit to Rhea’s school, every student had a photoshoot for their job applications. In contrast, talk of the CEE and *Gymnasium* came up only among groups of peers during breaks, during the extracurricular CEE preparation lessons, and after school. Obtaining information on the CEE and the *Gymnasia* and registering for the CEE were largely perceived to lie outside the scope of responsibilities of their schools, which was confirmed in various interviews with teachers and parents. As Maya’s teacher explained:

I do my thing and I believe that they can do that [CEE preparation] themselves . . . as I say that anyone who has the skills for the *Gymi* can prepare for themselves. (Interview, February 2022)

The predominance of the topic of apprenticeships is mirrored by many parents of the participants. For example, Nina’s mother describes her daughter’s school as a place where ‘they [students] should really just pull through these secondary school years’ and where the preparation for *Gymnasium* does not really play a role as ‘it’s not quite their job’. For Madison, this situation meant that her plan to transfer to *Gymnasium* was being overshadowed by the focus on VET at her secondary school:

*Gymnasium* should be a priority in my life now, but the whole week I’m at school, and I visit their CEE preparation course only one day a week. At school, we only talk about apprenticeships and, overall, the professional world. And that’s why I’m somehow attracted more to that now. To finding an apprenticeship and so on. My teacher also tells me all the time: ‘Apply for apprenticeships!’. But I know there’s also the *Gymnasium*, and it interests me a lot. So, I just do what I can. (Interview, February 2022)

Madison was the only student in this study that did not visit a private CEE preparation course. According to her, students who visit private preparation courses only do so because their parents want them to go there. Some students regarded the school courses as a supplement to their fee-based courses and tutors. One teacher told Lara that some students continue working on their teaching materials from their costly private CEE courses in their secondary schools’ CEE course; a practice that hints at the entanglements between private supplementary education and public schooling.

Students mentioned a contradiction between their educational aspirations and the reality they faced during their everyday life at school, where the CEE and *Gymnasium* played a subordinate role. Maya and many other study participants accepted the responsibility placed on them by booking private preparatory courses or by ‘just [doing] what I can’, as Madison explained. However, some students voiced resentment that had risen out of this contradiction. For example, Céline openly expressed her frustration at her school one morning in the weekly class-meeting:

So, they [classmates] always said ‘I have to write an application and so on’ [instead of continuing on school work], and then I was like: ‘Come on, write it finally, I want things to move forward for once!’ Because our teacher can work much better knowing that everyone has found an apprenticeship. (Interview, February 2022)

When Céline openly exhorted her class ‘to move forward for once’, she was confronted with the angry reactions of her classmates blaming her that she would ‘consider herself superior’. Céline had come to terms with her perceived lack of support at school for the CEE and concluded that ‘everything at school is your own responsibility’; here, ‘everything’ corresponds to Céline’s educational priority: The transition to *Gymnasium*.

The structure of Zurich’s education system contributes to the formation of a governmentality that shifts most of the responsibility for preparing for the CEE onto the individual students who want to continue their education at *Gymnasium*. This is also shown within discourses cultivated at this transition that render CEE preparation as a mainly personal matter that boils down to individual responsibility and personal decisions to get private help. Then, what does it mean for children such as Madison saying that they ‘just do what [they] can’? How are children governed and how do they govern themselves?

### 5.2. Children’s technologies of the self

#### 5.2.1. Disciplining and caring for the self by using private CEE preparation

Most of the study participants were governed by an overarching imperative to search for private CEE support. Visiting a private preparation course or tutor was considered a necessity, because many students believed that the CEE preparation at their secondary school did not prepare them well enough for their educational aspirations. Many children explained that their wish for private CEE preparation emerged from them and not their parents’ ambitions. As Eva’s Mother confirmed during an interview:

I have not researched this myself. Simply because Eva told me, ‘Mummy, that’s never enough! Everyone goes to a private preparation’. So, there’s already this pressure from society saying: ‘This. Is. Never. Enough’. (Interview, February 2022)

While some students had to convince their parents to pay for costly private preparation courses, others were denied this option. Caliyana, for example, told Lara during their first encounter that she had found her CEE preparation course at the private initiative ‘Chance4You’ during her online research and then contacted them directly via e-mail. At Chance4You, Caliyana and other students from families with low socioeconomic status received free CEE preparation lessons. Previously, Caliyana had researched various private preparation schools and only then terrifyingly learned that ‘education can also cost something’, as her teacher told Lara. Private preparatory CEE lessons also sometimes represented a financial burden for parents who bought their children a private course. Hence, Eva and Nina’s parents decided to share their daughters’ private CEE lessons to save money.

What connects Caliyana, Eva and Nina’s stories is that they were driven by a need for private CEE support. This pressure to look for private help when preparing for the CEE was experienced by most study participants. Students generally agreed that the ones who have ‘prepared the best’ would pass the test. Hence, for many children, attending a private CEE course appeared as the only logical ‘choice’ to succeed at the selective CEE, as Maya explained:

If they [students] want to make it, students will go to private preparation courses. The system exists, and it doesn’t adapt to how the students are; the students have to adapt to it. And if they want to make it, if they want to be sure that they will make it, then they go to a private preparatory school. (Interview, January 2022)

Maya’s observations illustrate the neoliberal governmentality engrained in the educational transition to *Gymnasium*: Students that want to make it to *Gymnasium* must conform to the ‘system’, which expects them to accept the responsibility placed on them by adhering to an idealized student subjectivity fit for the CEE. Of course, students have always had to ‘adapt’ to the education system. Yet, we argue that students in year eight and nine find themselves in an especially challenging situation due to the very structure of Zurich’s education system that sidelines the educational trajectory to *Gymnasium* from secondary school as demonstrated in the last chapter. As a consequence, children are forced to act as self-responsible, self-optimizing individuals who make ‘smart choices’ in seeking private supplementary learning support (cf. Bradbury 2019). Further, by stating that ‘if they want to make it . . . then they go to a private preparatory school’, Maya points at the reaffirming relationship between secondary school and private test preparation promoted by the competitive situation at this transition. This relationship rests in a neoliberal governmentality as it privatizes educational success at the transition to *Gymnasium*.

Visiting a private CEE preparation course can be seen as a technique that allows the self to adapt to this ‘existential norm’ (Laval and Dardot 2013, 3) within this neoliberal governmentality at the transition to *Gymnasium*. As our data suggests, children instrumentalized private CEE preparation to discipline and care for the self as entrepreneurial selves (c.f. Bröckling 2016). Private courses, in contrast to state-funded courses, were regularly described as ‘serious’, ‘strict’, ‘professional’, and ‘personalized’ – ‘a proper preparation’, as Sam assessed his private course at Chance4You when comparing it to the one he had attended at his secondary school. Generally, participants described a ‘good’ preparation as ‘intensive’, ‘strict’, ‘structured’ and hence taken ‘seriously’. After all, you do not attend a preparation course ‘for fun’, as Eli explained. Children often pointed at the way private CEE courses ‘translate’ the knowledge needed for the CEE into ‘digestible pieces’ – a service that the children found beneficial.

While students used private CEE preparation to discipline the self, they also saw it as a necessity to care for the self: to feel ‘mentally supported’ as Caliyana put it. This duality of simultaneously disciplining and caring for the self is visible in Eli’s description of the teachers at his private preparation course:

They really put so much effort into it. There are strict rules and they say: ‘You are here to pass the CEE’. They are also really helpful: They open up options for us that I don’t have with normal teachers and are really there for us who want to pass the CEE. (Interview, February 2022)

On the one hand ‘there are strict rules’, on the other hand Eli regards his private preparation teachers as being ‘really there’ for their students and caring about them: he feels that they want their students to pass the CEE. Students often characterized their private teachers as demanding but also as highly supportive, and caring, some of them even offering help during holidays. A headmaster at a secondary school framed this support that Eli and others got at their private CEE preparation as ‘buying yourself free from responsibility’. This mental and academic support of for-profit CEE preparation is an expression of the neoliberal governmentality at this transition as it allows to partly alleviate stress and outsource the individualized responsibility this transition entails in exchange for money.

While students are governed by the pressure to use private CEE support, they simultaneously govern other students by cultivating an exclusionary discourse that viewed only the students who use private preparation as ‘taking CEE preparation seriously’. According to some students, secondary school’s free preparation courses were only visited by ‘the ones who aren’t that serious about it’, as Hannah put it. Similarly, Eva knew early on that she would need ‘something extra to the school course’ and told Lara that ‘of course, you’ll also have to go private’. One evening, as Lara accompanied Eva and Nina on a visit to Mr. Pine, their shared private tutor, they talked about Emma, a girl at their school who only irregularly joined Mr. Pine’s CEE preparation lessons:

‘Emma isn’t here today, so we can really move up a gear’. Eva tells Nina. Nina nods, looks at me and explains that Emma would have had to choose between ‘going to the restaurant with her family’ and ‘visiting Mr. Pine’. Now she just decided to go to the restaurant, Nina tells me and Eva adds reproachfully that ‘she just doesn’t take it that seriously’. (Fieldnotes, February 2022)

While Nina and Eva were both governed by the pressure to use private CEE support, they simultaneously governed other students, such as Emma through an exclusionary discourse that distinguishes between the students who ‘take the CEE seriously’ and those who allow ‘free choice’ dissolve ‘into a failure of the self to adequately take up the burdens of being the appropriate(d) subject of individualism and responsibilization’ (Davies and Bansel 2007, 256). These stories not only illustrate how students use private preparation as forms of self-techniques, but they also show how private preparation works as an exclusionary tool within this neoliberal governmentality.

#### 5.2.2. Self-discipline and self-reflection through study plans and tracking apps

The ways students are governed ‘with the help of others’ are complemented by more subtle and intimate ways of governing themselves ‘by their own means’ (Foucault 1988, 18). In fact, for many children, ‘taking CEE seriously’ meant more than attending a private preparation course. It meant ‘sacrificing’ a part of their lives, as Nina said. For most, this sacrifice involved giving up hobbies that they would usually enjoy; for others, it also impacted their friendships. Eli, for example, decided to cut down on meeting a friend of his to save more time for studying. A few weeks later, Eli told Lara that he had broken off contact with his friend ‘for good’: ‘I don’t want her to distract me. I have to focus on what I want to achieve’. For Aiza, preparing for the CEE resulted in spending less time with her family. A few weeks after the CEE, Aiza explained: ‘When I spent an hour with the family, I always thought I’d rather do an hour of math in my room’. Disconnecting with friends and family for the assumed benefit of success at the CEE mirror the effects of this transition’s neoliberal governmentality that can reduce well-being and increase loneliness (c.f. J. Becker, Hartwich, and Haslam 2021).

Besides subordinating leisure time, friends, and family to study time, most children followed self-made study plans, lists, and tracking apps to stay focused. For many, creating daily lists and plans had become a routine and a way ‘to stay focused’, as Eva explained. When Lara spent a morning with Eva during the holidays, she introduced her to her ways of planning the days before the CEE by creating daily lists. Additionally, she wrote entries in her diary reflecting on her mood, what she had done that day and what she could improve. In one such entry that she shared with Lara, she wrote that she was happy that day and had enjoyed the nice weather but should ‘do more for school’, ‘spend less time on the phone’, ‘read more’, and ‘practice’. That morning, Eva also showed Lara around in her room, which was decorated with drawings, posters, and photographs. She drew Lara’s attention to a letter light box displaying the words: ‘#DON’T GIVE UP’, telling her that this was currently her ‘motto’. Eli also reflected on his behavior, emotions, and daily accomplishments by using a tracking app on his phone and putting down brief notes such as ‘studied for CEE, went to bed early’ or ‘solved a mock exam, had long day at school’. The app showed how long he had adhered to his plans and would rewind the line of progress if Eli quit studying.

For many students, using lists, apps, diaries, and motivational quotations had more profound meaning than simply staying focused. In fact, Eva and Eli illustrated, they are a tool for self-reflection and an expression of one’s own willingness, discipline, and even self-surveillance. According to Eli, when he had studied for the CEE the previous year without success, he had not been ‘mentally ready’ for this preparation time and, hence, had not kept to his plans. Now, he described himself as ‘independent’, ‘ambitious’, and ‘willing’ and saw studying for the CEE as part of his self:

I have the feeling that it’s completely normal: When I get home, I study for the CEE because it has started to feel like it’s become part of me after all this time. (Interview, January 2022)

Similarly, Caliyana, who followed intensive daily study plans (see below), reflected on her current self as ‘more focused’ than she would have been previously. According to her, she had stopped going out on Friday evenings and would ‘get upset’ if she rose after 9 am on weekends or during the holidays as she would ‘oversleep time that I could use for studying’. On Monday, 20 December, during the Christmas break, Caliyana’s daily study plan included the following points:

07:30-08:00: Preparation

08:00-09:00: English-Presentation

09:10-10:00: English-Presentation

10:10-11:00: English-Presentation

11:10-12:00: Finish French Theory

13:00-14:00: Repeat French Theory

14:10-15:00: Work on French Workbook

15:10-16:00: Work on French Workbook

16:10-17:00: Work on French Workbook

17:10-18:00: Repeat German Theory

18:10-19:00: Work on German Workbook

19:10-20:00: Repeat French Vocabulary

20:00-21:00: Break

21:00-22:00: Work on Math Workbook

22:15-23:00: Work on Math Workbook

00:00 –?: One Episode of Gilmore Girls

? – 07:00: Sleep (Own translation of a photograph, December 2021)

Student’s focus on self-management through planning and following their rigid CEE preparation schemes illustrate the neoliberal governmentality that children navigate at this transition. This is evident in our finding that the content of their studies was largely overshadowed by their focus on extensive self-management exercises as presented in this chapter. Lara witnessed students upholding an image of an idealized self as independent, ambitious, persistent, and even self-disciplined while simultaneously talking of exhaustion, pressure, burnout, and anxiety (cf. Davidson 2008).

This neoliberal focus on optimizing their preparation for the CEE through a diverse set of self-techniques represented a balancing act: Studying too much resulted in feeling ‘burnt out’ (Caliyana), whereas studying too little caused the students to feel ‘lazy and stupid’ (Aiza). Aiza and Caliyana, who drafted similar 16-hour study plans, both explained several times that they had ‘lost their motivation’ due to their schedule. They knew that their study plans would not work out, but they still adhered to them. Aiza and Caliyana were not the only students struggling with their CEE preparation. Some students developed strategies for calming down in stressful situations by, for example, humming to her pet (Eva) or sitting over the books even longer, ‘just to get the feeling that I’m doing enough’ (Eli).

Eventually, however, the children’s preparation time for the CEE came to an end. Eight months after the CEE, when Lara caught up with Caliyana and others, Caliyana looked back on the months of CEE preparation in disbelief, explaining that she had taken the CEE preparation too far:

I was under a lot of pressure. And I had taken it too far. And somehow, I thought that I had to do exactly that because otherwise I couldn’t be happy. And others wouldn’t be happy with me either. And that was completely wrong . . . it doesn’t matter now that I didn’t pass this exam, because I’m still good at school and I’m still happy. (Interview, November 2022)

Caliyana did not pass the CEE. Yet, contrary to her former assumptions, she was still happy. Whether or not students passed the CEE, the participants expressed that a major burden appeared to have fallen away – or, as Aiza put it, created room ‘to live life again’.

## 6. Conclusion

Research on children’s experiences in neoliberal educational contexts have tended to emphasize the techniques used to govern children in education. For example, these techniques have been shown within education practices (e.g. Hangartner et al. 2022; Kelly 2019; Morrin 2018), education policies (e.g. Bradbury 2019; Phillippo et al. 2020), economic restructuring affecting education (e.g. Holloway and Kirby 2020; Mitchell 2018; Pimlott-Wilson 2019), the role of gender in school (e.g. Davidson 2015; Youdell 2006) and educational pressure to maintain or ascend social status (e.g. Davidson 2008; Katz 2018). While these studies present valuable insights into education’s ‘technologies of domination’ of students (Foucault 1988), the personal and intimate ways children develop as a response to these ways of being governed have taken a back seat. Yet, studying these very ways children develop to govern themselves in educational contexts allows for a more holistic and nuanced view of the of the workings of neoliberal power mechanisms in education (Lemke, Krasmann, and Bröckling 2000, 30). Following the later work of Foucault discussed previously, these mechanisms of power are best expressed in the interactions ‘between the technologies of domination of others and those of the self’ (1988, 19).

Building on this theorizing, we demonstrated the reciprocal dependencies between children’s technologies of the self and the education system’s structures of domination over its students (Lemke, Krasmann, and Bröckling 2000; von Felden 2020). As we have shown, children at the educational transition to *Gymnasium* developed their own ‘technologies of the self’ (Foucault 1988) that revealed new insights into the transition’s neoliberal governmentality. Conversely, children’s ‘technologies of the self’ appeared as an integral part of the educational transition’s very structures of domination. Our analysis demonstrated that students who aspire to make it to *Gymnasium* after year eight or nine feel forced to comply with a neoliberal governmentality that grows out of the very structure of Zurich’s education system. This finding opens up possible pathways for systemic change and resisting neoliberal developments within it (c.f. Tett and Hamilton 2019).

While acknowledging the contributions of existing scholarship, we recognize the responsibility to assume perspectives that focus on children’s intimate ways of navigating education systems. Earlier research has called for granting children a more active voice in research on education (Holloway et al. 2010; Nguyen, Cohen, and Huff 2017). Focusing on children’s technologies of the self serves as a pathway to answer and expand these very calls. This conceptual approach enables researchers to identify the often-invisible entanglements that children have with the education system they navigate. Our paper gives additional weight to this reasoning, as we have shown the extent to which a selective education system can interfere with children’s daily lives. This line of inquiry thus not only furthers debates in children’s geographies and governmentality studies but also provides conceptual prospects to stay close to children’s realities.

## Notes

1 Anonymity has been maintained for all participants.

2 The first author conducted the ethnography and analysis of data and wrote this paper collaboratively with the second author.
